# Royal Jelly Plus Coenzyme Q10 Supplementation Enhances High-Intensity Interval Exercise Performance *via* Alterations in Cardiac Autonomic Regulation and Blood Lactate Concentration in Runners

**DOI:** 10.3389/fnut.2022.893515

**Published:** 2022-06-23

**Authors:** Aleksandr N. Ovchinnikov, Anna V. Deryugina, Antonio Paoli

**Affiliations:** ^1^Department of Sports Medicine and Psychology, Lobachevsky University, Nizhny Novgorod, Russia; ^2^Department of Physiology and Anatomy, Lobachevsky University, Nizhny Novgorod, Russia; ^3^Department of Biomedical Sciences, University of Padua, Padua, Italy

**Keywords:** royal jelly, coenzyme Q10, athletic performance, running, autonomic nervous system, heart rate variability, energetic metabolism, lactate

## Abstract

**Purpose:**

This study aimed to examine whether oral royal jelly (RJ) and coenzyme Q10 (CoQ10) co-supplementation could improve high-intensity interval exercise (HIIE) performance in runners, reducing exercise-induced lactic acidosis and decreasing elevated sympathetic tone following exercise.

**Methods:**

Thirty regional-level runners (age: 19 ± 1 years; height: 173 ± 2 cm; body mass: 68.9 ± 2 kg; body mass index: 23.1 ± 1 kg/m^2^) were randomly allocated to receive either 400 mg of RJ and 60 mg of CoQ10 (RJQ) or matching placebo (PLA) once daily for 10 days. Exercise performance expressed as time taken to complete HIIE was evaluated at baseline, and then reassessed at day 10 of intervention. HIIE protocol applied to the runners included three repetitions of 100 m distance at maximum possible speed interspersed with 45 s of recovery periods. Indices of heart rate variability and blood lactate concentration were also measured before and immediately after HIIE in each group.

**Results:**

HIIE performance significantly improved in RJQ group (*p* = 0.005) compared to PLA group. Blood lactate levels and sympathetic influence on the heart were significantly lower both before and after the HIIE in athletes who received RJQ (*p* < 0.05) compared to PLA. Regression analysis showed that oral RJQ administration for 10 days was significantly associated with reductions in HIIE-induced increases in blood lactate concentration and enhanced cardiac parasympathetic modulation following exercise compared to PLA. Principal component analysis revealed that runners treated with RJQ are grouped by the first two principal components into a separate cluster compared to PLA. Correlation analysis demonstrated that the improvements in runners’ HIIE performance were due in significant part to RJQ-induced reduction of increment in blood lactate levels in response to exercise in combination with a more rapid shift in autonomic activity toward increased parasympathetic control early at post-exercise.

**Conclusion:**

These findings suggest that RJQ supplementation for 10 days is potentially effective for enhancing HIIE performance and alleviating adverse effects of increased intramuscular acidity and prolonged sympathetic dominance following intense exercise.

## Introduction

It is crucial to take into consideration any athlete’s fatigue state and/or performance responses to the training load. Among the methods available for diagnosing non-functional overreaching or overtraining syndrome, the heart rate variability (HRV) is widely used because its alterations depend largely on changes in cardiac autonomic regulation which continuously attempts to adapt cardiovascular function to exercise and training (1–4). The decrease in autonomic nervous system (ANS) activity during intensive training is correlated with the loss in exercise performance, and the rebound in ANS activity with its increase (5). In addition, scientific data have shown that ANS recovery is more rapid in highly trained than in trained subjects following high-intensity exercise (6).

Blood lactate concentration is also one of the most often measured parameters during performance testing of athletes (7). Although lactate is now recognized as an important metabolic intermediate and signaling molecule mediating exercise adaptations and interorgan communication (8–13), its accumulation in muscle and blood at higher levels can lead to acidosis and negatively affect force and power generation, resulting in a reduction in sports performance due to inability to maintain the exercise intensity needed for ultimate success (14–16).

The two major approaches used to enhance fatigue resistance and, thus, sports performance are regular training and various nutritional interventions, and the interactions between them have been recognized (17, 18). Royal jelly (RJ), which is secreted by the cephalic glands of honey bees (*Apis mellifera* L.) and used as food for young larvae and queen bees, contains many nutrients including vitamins, minerals, fatty acids, carbohydrates, and proteins/amino acids (19). Some ingredients contained in RJ have been suggested to have a potential for increasing ANS activity and energy metabolism in skeletal muscles (20–22). In addition to RJ supplementation, our line of evidence indicates that coenzyme Q10 (CoQ10), which in turn plays a key role in supplying energy to all cells and taking part in redox reactions within the electron transport chain at the mitochondrial level (23–25), may yield synergies toward fatigue resistance and the improvement of athletic performance (26).

Our aim was to conduct a 10-day, randomized, double-blind, placebo-controlled trial to examine whether oral RJ and CoQ10 co-supplementation could improve high-intensity interval exercise (HIIE) performance in runners, reducing exercise-induced lactic acidosis and decreasing elevated sympathetic tone following exercise. We hypothesize that RJ plus CoQ10 (RJQ) supplementation is able to enhance HIIE performance, which can be associated with RJQ-induced neurohumoral and metabolic changes, in particular with a more rapid shift from parasympathetic to sympathetic going from rest to exercise and vice versa, as well as with reducing the increment in blood lactate concentration in response to exercise.

## Materials and Methods

### Subjects

Thirty male athletes were recruited from a single sports training center (Nizhny Novgorod, Russia) by advertising directly to coaches. Subjects were included in the study if they had at least sport category «Candidate Master of Sports» or sport rank «Master of Sports of Russia» in accordance with the Unified Russian Sports Classification System. Exclusion criteria were the use of any antioxidant supplements, including RJ or/and CoQ10, and/or chronic use of any medication. Two weeks before intervention, the above-mentioned criteria were excluded by coaches’ interview, and all subjects were included in the study. Before any procedures, informed written consent was obtained from each of the study participants. At the end of the first screening visit, subjects were randomly assigned to the groups: RJ plus CoQ10 (RJQ; *n* = 15; age: 19.40 ± 1.50 years; height: 172.13 ± 2.07 cm; body mass: 68.53 ± 1.96 kg; body mass index: 23.15 ± 0.93 kg/m^2^) or placebo (PLA; *n* = 15; age: 19.13 ± 1.06 years; height: 173.00 ± 2.20 cm; body mass: 69.20 ± 2.01 kg; body mass index: 23.13 ± 1.02 kg/m^2^). The study protocol was approved by the Bioethics Committee of Lobachevsky University (approval number: 43). The study was conducted in accordance with the guidelines laid down in the Declaration of Helsinki (27).

### Study Design

This study is a 10-day, randomized, double-blind, placebo-controlled trial. After signing the informed consent and 2 weeks before intervention, the participants were referred to the Integral Human Health Laboratory of the Faculty of Physical Education and Sport of Lobachevsky University. During the visit, the athletes underwent a medical screening to ensure eligibility for the study and food interview to gather information on the subjects’ dietary habits.

Thirty runners were randomized in a double-blind fashion in a 1:1 ratio and assigned to receive either RJQ (*n* = 15) or PLA (*n* = 15) once daily for 10 days. To allocate subjects, we applied a computer-generated list of random numbers. Participants were instructed to avoid taking any additional supplements containing RJ and/or CoQ10, as well as any antioxidant supplements during the study. Participants were also instructed to have breakfast 1 h before the training sessions and refrain from consuming alcohol and caffeinated beverages for at least 24 h before exercise testing. Adherence to study medications was assessed daily by a qualified dietician with a face-to-face interview. Athletes randomized to the RJQ group received supplementation daily consisting of active ingredients (60 mg of CoQ10 and 400 mg of RJ) and excipients (10 g of honey). Athletes randomized to PLA group received supplementation daily without active ingredients and containing only excipients (10 g of honey). Both active and placebo supplements were identical in size, color, opacity, shape, presentation, and packaging. The substances were taken sublingually in the morning. CoQ10 was manufactured and donated by OJSC «Kstovo Experimental Pilot Protein-Vitamin Concentrates Plant» (Kstovo, Russia). RJ and honey were manufactured and donated by Federal Beekeeping Research Center (Sochi, Russia). Participants were asked to consume RJQ supplementation 60 min before the training session. This is a sufficient time for RJ-induced activation of 5′-AMP-activated protein kinase (AMPK) and, perhaps, for total coenzyme Q10 level in skeletal muscle cells to become elevated to get an observable ergogenic effect (21, 28–30). One day before the start of the intervention, runners underwent their specific HIIE on a 400 m outdoor track of the stadium. HIIE protocol applied to the runners included three repetitions of 100 m distance at maximum possible speed interspersed with 45 s of recovery periods. Exercise testing was performed in the morning. Subjects were examined before and immediately after HIIE for HRV measures and blood sample collection, followed by determination of lactate levels. Athletes kept a typical training routine, which was the same for all participants. Runners took part in the study in a period away from the competition phase: workouts had a daily schedule and training sessions included exercises mainly aimed at increasing speed endurance. After 10 days, subjects repeated the HIIE and, simultaneously, HRV indices and blood lactate levels were reassessed before and immediately after the exercise.

### Investigational Substances

Exogenous CoQ10 is a microbiological synthesis product synthesized at OJSC «Kstovo Experimental Pilot Protein-Vitamin Concentrates Plant» according to the technology developed at the Research Institute «Sintezbelok» of the Russian Academy of Sciences and Research and Production Association «Vitaminy». According to TU—op. 64-12-125-90, CoQ10 is a yellow-orange crystalline powder with the following properties: molecular formula—C_59_H_90_O_4_, molecular weight—863.36 g/mol, and melting point—47.5–49.0^°^C.

Native fresh royal jelly was extracted in the Federal Beekeeping Research Center. Organoleptic and physicochemical properties of royal jelly complied with the requirements of interstate standard approved by the Interstate Council for Standardization, Metrology and Certification. In accordance with the above-mentioned standard, native fresh royal jelly contained a minimum of 5% decenoic acids (in royal jelly containing no water) where the largest proportion of these acids belonged to 10-hydroxy-2-decenoic acid. Honey was also extracted in the Federal Beekeeping Research Center.

The tested combination is a native fresh royal jelly plus coenzyme Q10 dissolved in a carrier lipid. To determine doses and timing, we were guided by recommended dosages and timing of interventions when using approved biologically active food supplements, in particular «Apitonus» (10–20 g per day) and «Kudesan» (1–2 ml per day). «Apitonus» is a combination of native fresh royal jelly and honey mixed in a 2:100 ratio, respectively. «Kudesan» is a water solution of solubilized coenzyme Q10 (30 mg/mL).

### Measurement of Exercise Performance

The HIIE protocol, applied to the runners, consisted of three repetitions of 100 m distance at maximum possible speed, interspersed with 45 s of recovery time. The aim of the test was to complete each set in the shortest possible time. Results were determined by the time taken to complete each set with the subsequent calculation of mean. Timing was recorded using a stopwatch from the start signal until the runner crossed the finish line.

### Analysis of Heart Rate Variability

To test general autonomic reactivity using a standard method, heart rate and HRV changes of all participants were measured before and immediately after the exercise. All subjects were instructed to refrain from ingesting beverages containing caffeine or alcohol for 24 h prior to the testing. ECG signal was recorded by a hardware-software complex «Poly-Spectrum-Rhythm» (“Neurosoft,” Ivanovo, Russia) for 5 min in the supine position in a quiet room with normal lighting and room temperature. The participants were asked to remain silent without deep breathing, speaking, or moving at all. The data collection was always performed by the same researcher, who maintained the same conditions for, and gave identical instructions to, all the participants. The signal processing was performed using Neurosoft^®^ HRV analysis software with time and frequency domain analysis. The methodological criteria that were applied were those proposed by the Task Force of the European Society of Cardiology and the North American Society of Pacing and Electrophysiology (31).

In the HRV time domain, the mean RR interval, the SD of NN intervals, the square root of the mean squared difference of successive RR intervals (RMSSD), and the percentage of consecutive RRs that differed by more than 50 ms each were obtained.

A Fast Fourier Transform of the RR signals was used for the HRV frequency domain analysis. The spectral response provided by the system was broken down into three bands: high frequency (0.15–0.4 Hz), low frequency (0.04–0.15 Hz), and very low frequency (0.003–0.04 Hz).

Baevsky’s Stress Index (SI) (32) was calculated according to the formula:


$$S\,I=\frac{A\,M_{o}}{2\times M_{o}\times M_{x}\,D\,M_{n}}$$

where the mode (*M*_*o*_) is the most frequent RR interval expressed in seconds. The amplitude of mode (*AM*_*o*_) was calculated, using a 50 ms bin width, as the number of the RR intervals in the bin containing the *M*_*o*_, expressed as a percentage of the total number of intervals measured. The variability is reflected in *M_*x*_DM_*n*_* as the difference between longest (M*_*x*_*) and shortest (M*_*n*_*) RR interval values, expressed in seconds. The SI is expressed as s^–2^.

### Measurement of Blood Lactate Concentration

Finger-prick blood samples were collected by a qualified phlebotomist before (at rest) and 7–8 min after the exercise. Lactate concentration was measured by a hand-held point-of-care device “StatStrip^®^Xpress™” (Nova Biomedical, Waltham, MA, United States) that utilizes 0.7 μL of blood and electrochemical test strips.

### Statistical Analysis

A descriptive statistical analysis was carried out for all study variables. Data are expressed as mean ± standard deviation (*SD*). The sample was checked for normal distribution using the Shapiro–Wilk test. Since all data were normally distributed, comparisons were made using paired Student’s *t*-test for within-group comparison analysis and using Student’s *t*-test for independent data for between-group comparison analysis. According to the pre-specified analysis plan, treatment effect was assessed as the mean change in each parameter from pre-exercise to post-exercise for each intervention group. Statistical comparison between the treatment effects on exercise-induced changes in HRV indices and blood lactate concentration was made by regression analysis using a following model:


$$Y=\beta_{0}+\beta_{1}\times D\,u\,m\,m\,y+\varepsilon$$

where Y—response variable; Dummy—dummy variable between 0 and 1 depending on the supplement (0 = PLA, 1 = RJQ); β_0_ and β_1_—estimated regression parameters; and ε—modeling error.

The regression model made it possible to determine whether the mean changes in each parameter between the RJQ group and PLA group under HIIE conditions were statistically significant. In addition, principal component analysis (PCA) was applied. PCA made it possible to take into account the variances of all RJQ-dependent variables in the subsequent data analysis in order to establish a causal relationship between formed factor, consisting of HRV indices and blood lactate, and HIIE performance, since PCA is based on the transition from the space of correlated variables to the space of orthogonal variables (principal components). Thus, the use of PCA, on the one hand, solved the arisen problem of multicollinearity of the RJQ-dependent variables. Statistical relationships between exercise-induced changes in HRV indices and blood lactate concentration at day 10 of intervention were evaluated using Pearson’s correlation coefficient (*R*). On the other hand, PCA provided an opportunity to deviate from direct modeling of HIIE performance, since the inclusion in model specification exclusively investigated parameters and ignoring the rest of the regressors would lead to correlation between model error and independent variable (endogeneity problem). In this regard, we attempted to indirectly explain variation in the completion time by means of explaining variation in changes of the RJQ-dependent variables. Since the set of the analyzed parameters has different units of measurement, it is logical to assume that principal component 1 (PC1) will include variables with the highest sample variance as with the highest weighting coefficients. Thus, in order for the PCA to be applicable, the RJQ-dependent variables were normalized using the following formula:


$$x_{i_{j}}=\frac{a_{i_{j}}-{\bar{a}}_{i}}{S\,E\,M\,\left(a_{i}\right)}$$

where *a_i__j_*−j-th observation of the i-th variable; ${\bar{a}}_{i}-$mean of the i-th variable; and *SEM*(*a*_*i*_)−standard error of the mean of the i-th variable.

For all analyses, a *p*-value < 0.05 was considered statistically significant. All statistical analyses were performed using RStudio, version 1.3.1093 for macOS (RStudio, PBC).

## Results

**FIGURE 1:**
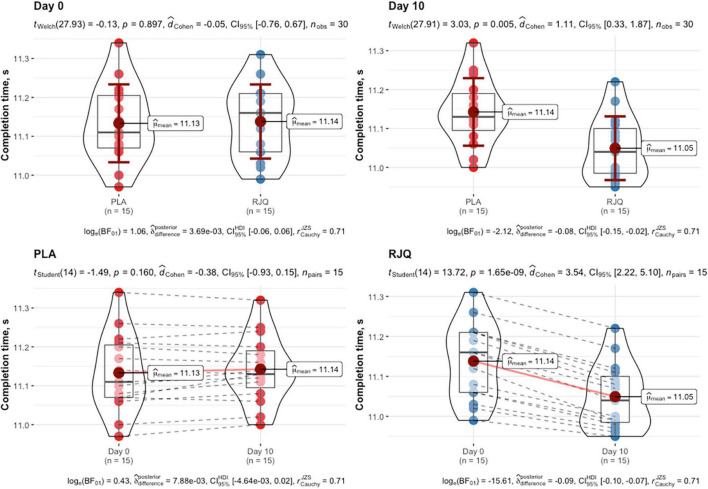
HIIE performance expressed as time to complete exercise at baseline and day 10 of intervention between randomization arms. Data are given as means ± *SD* and compared by paired Student’s *t*-test for within-group comparison analysis and by Student’s *t*-test for independent data for between-group comparison analysis. *n* = 15 per group.

All the recruited subjects successfully completed the study, and no side effect from RJQ intake was reported. Measurements of HIIE performance (i.e., completion time) in runners at baseline and day 10 of intervention are presented in Figure 1.

The RJQ supplementation significantly decreased the time taken by runners to complete HIIE, compared to PLA (RJQ group: from 11.14 ± 0.10 s to 11.05 ± 0.08 s; PLA group: from 11.13 ± 0.10 s to 11.14 ± 0.09 s). At day 10 of the intervention, the sympathetic parameter SI and blood lactate levels were significantly lower both before and after the HIIE in RJQ group compared to PLA group (Table 1).

**TABLE 1:** Heart rate variability indices and blood lactate concentration before and immediately after high-intensity interval exercise at baseline and day 10 of intervention between randomization arms.

Physical	Day 0		Day 10	
Performance variables	Pre-exercise	Post-exercise	Pre-exercise	Post-exercise
**HR, bpm**				
RJQ PLA	69.60 ± 5.36 67.33 ± 4.88	102.87 ± 6.63* 101.20 ± 5.03*	64.00 ± 3.25* 66.67 ± 3.79^§^	96.27 ± 5.99^#†^ 101.93 ± 5.78^#§^
**RRNN, ms**				
RJQ PLA	885.53 ± 37.50 896.00 ± 33.74	597.73 ± 25.11* 606.40 ± 32.60*	920.93 ± 4.86* 895.40 ± 34.28^§^	635.93 ± 17.67^#†^ 605.07 ± 41.43^#§^
**SDNN, ms**				
RJQ PLA	85.40 ± 3.79 87.40 ± 3.27	21.80 ± 6.16* 22.60 ± 6.17*	94.73 ± 2.02* 88.67 ± 3.20^§^	38.07 ± 4.83^#†^ 22.27 ± 5.57^#§^
**RMSSD, ms**				
RJQ PLA	80.13 ± 3.07 81.20 ± 2.62	8.87 ± 2.56* 9.40 ± 2.47*	85.80 ± 1.93* 80.87 ± 3.64^§^	22.93 ± 4.92^#†^ 9.27 ± 2.28^#§^
**pNN50, %**				
RJQ PLA	24.13 ± 2.00 24.60 ± 1.76	2.33 ± 1.63* 2.47 ± 1.60*	26.60 ± 1.92* 24.73 ± 2.25^§^	7.93 ± 1.79^#†^ 2.27 ± 1.22^#§^
**HF, ms^2^**				
RJQ PLA	3332.53 ± 22.63 3340.87 ± 26.19	103.53 ± 5.55* 104.07 ± 5.11*	3396.40 ± 4.08* 3331.00 ± 22.79^§^	848.87 ± 10.83^#†^ 103.80 ± 6.04^#§^
**LF, ms^2^**				
RJQ PLA	2807.93 ± 18.96 2812.73 ± 21.64	412.87 ± 9.43* 416.80 ± 7.95*	2893.73 ± 5.01* 2800.00 ± 15.15^§^	1094.73 ± 12.52^#†^ 419.20 ± 13.37^#§^
**VLF, ms^2^**				
RJQ PLA	1038.47 ±6.80 1039.87 ± 6.95	304.27 ± 6.13* 304.80 ± 7.08*	1090.27 ± 3.43* 1037.00 ± 6.74^§^	618.27 ± 6.33^#†^ 304.53 ± 7.33^#§^
**SI, a.u.**				
RJQ PLA	61.93 ± 9.44 61.87 ± 8.88	739.33 ± 89.03* 738.60 ± 92.55*	44.47 ± 8.48* 62.67 ± 9.96^§^	396.33 ± 63.40^#†^ 731.87 ± 89.52^#§^
**Lactate, mmol/L**				
RJQ PLA	1.20 ± 0.05 1.19 ± 0.04	16.55 ± 0.62* 16.52 ± 0.73*	1.13 ± 0.07* 1.20 ± 0.04^§^	14.13 ± 0.69^#†^ 16.62 ± 0.61^#§^

Data are given as means ± SD and compared by paired Student’s t-test for within-group comparison analysis and by Student’s t-test for independent data for between-group comparison analysis. n = 15 per group. *Significantly different from pre-exercise value at day 0 (p < 0.05); ^#^significantly different from pre-exercise value at day 10 (p < 0.05); ^†^significantly different from post-exercise value at day 0 (p < 0.05); ^§^significantly different from RJQ group (p < 0.05).

*HR, heart rate; RRNN, mean RR normal-to-normal intervals; SDNN, standard deviation of normal-to-normal intervals; RMSSD, root mean square of successive RR intervals differences; pNN50, percentage of successive RR intervals that differ by more than 50 ms; HF, absolute power of the high-frequency band; LF, absolute power of the low-frequency band; VLF, absolute power of the very-low-frequency band; SI, stress index; RJQ, royal jelly plus coenzyme Q10; PLA, placebo.*

The RJQ supplementation resulted in an increase in RRNN and decrease in heart rate, respectively. The parasympathetic parameters such as SDNN, RMSSD, pNN50, and HF power were significantly higher both at rest and following HIIE in athletes treated for 10 days with RJQ compared to PLA, consistent with an increase in parasympathetic reactivity at post-exercise. In relation to LF power and VLF power, participants who received RJQ also had greater values compared to PLA both at pre-exercise and at post-exercise.

To assess the effect of 10-day RJQ supplementation on HIIE performance (based on the observed completion time) through a combination of decreasing regulation strain (based on the observed HRV measures) and reducing lactic acidosis under HIIE conditions, further correlation and regression analysis using principal components were applied.

**FIGURE 2:**
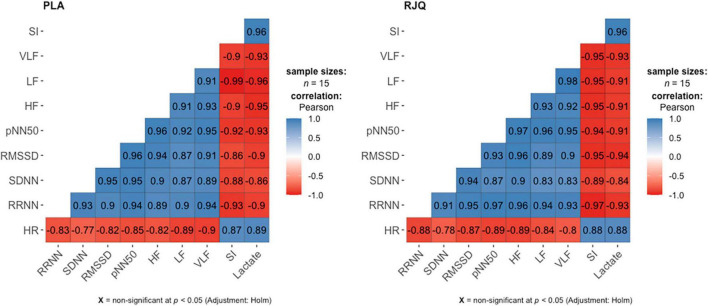
Correlation matrix for displaying the relationship between the changes in HRV parameters and blood lactate levels under HIIE conditions in both intervention groups. *n* = 15 per group. HR, heart rate; RRNN, mean RR normal-to-normal intervals; SDNN, standard deviation of normal-to-normal intervals; RMSSD, root mean square of successive RR intervals differences; pNN50, percentage of successive RR intervals that differ by more than 50 ms; HF, absolute power of the high-frequency band; LF, absolute power of the low-frequency band; VLF, absolute power of the very-low-frequency band; SI, stress index; RJQ, royal jelly plus coenzyme Q10; PLA, placebo.

A strong positive correlation between the mean changes in HRV indices and blood lactate concentration under HIIE conditions in both intervention groups was observed (Figure 2).

Regression analysis showed that oral RJQ administration for 10 days was significantly associated with reductions in HIIE-induced increases in heart rate, SI, and blood lactate concentration compared to PLA (Table 2).

**TABLE 2:** Modeling the effects of 10-day RJQ supplementation on changes in HRV parameters and blood lactate levels in response to high-intensity interval exercise.

Response variable (Y)	Intercept (β_0_)	Regression coefficient (β_1_)	Multiple R-squared
HR, bpm	35.2667***	–3.000**	0.2469
RRNN, ms	–290.333***	5.333	0.05655
SDNN, ms	–66.400***	9.733***	0.4504
RMSSD, ms	–71.600***	8.733***	0.4909
pNN50, %	–22.4667***	3.800**	0.241
HF, ms^2^	–3227.200***	679.667***	0.9965
LF, ms^2^	–2380.800***	581.800***	0.9948
VLF, ms^2^	–732.467***	260.467***	0.9931
SI, a.u.	669.200***	–317.330***	0.8497
Lactate, mmol/L	15.4133***	–2.4133***	0.8131

Data are presented as β_0_ (mean changes in athletes treated with PLA) and β_0_ + β_1_ (mean changes in athletes treated with RJQ). n = 15 per group. Significance level was set at ***p < 0.001 and **p < 0.01.

*HR, heart rate; RRNN, mean RR normal-to-normal intervals; SDNN, standard deviation of normal-to-normal intervals; RMSSD, root mean square of successive RR intervals differences; pNN50, percentage of successive RR intervals that differ by more than 50 ms; HF, absolute power of the high-frequency band; LF, absolute power of the low-frequency band; VLF, absolute power of the very-low-frequency band; SI, stress index; RJQ, royal jelly plus coenzyme Q10; PLA, placebo.*

The marked RJQ-induced reduction in SI following exercise is consistent with a withdrawal of sympathetic activity. Moreover, the more rapid shift in autonomic balance toward a dominant parasympathetic activity was reflected by the RJQ-caused increment in SDNN, RMSSD, pNN50, and HF power immediately after the HIIE. An important finding was that HIIE also induced a smaller decrease in LF power and VLF power in subjects who consumed RJQ compared to PLA. This finding may be considered as additional evidence that the LF power and VLF power are not specific for sympathetic activity.

**FIGURE 3:**
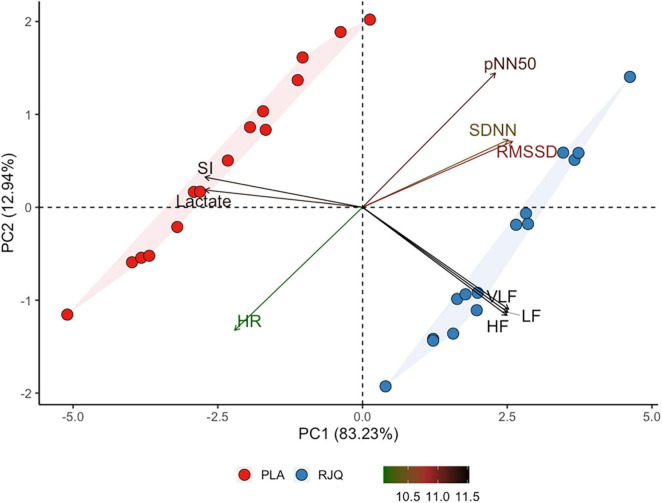
Principal component analysis applied to the changes in RJQ-dependent variables under HIIE conditions. *n* = 15 per group. PC1, principal component 1; PC2, principal component 2; HR, heart rate; SDNN, standard deviation of normal-to-normal intervals; RMSSD, root mean square of successive RR intervals differences; pNN50, percentage of successive RR intervals that differ by more than 50 ms; HF, absolute power of the high-frequency band; LF, absolute power of the low-frequency band; VLF, absolute power of the very-low-frequency band; SI, stress index; RJQ, royal jelly plus coenzyme Q10; PLA, placebo.

Principal component analysis revealed that PC1 describes almost 83.23% of the total variance of the changes in HR, SDNN, RMSSD, pNN50, HF, LF, VLF, SI, and blood lactate. In its turn, PC2, PC3, PC4, PC5, PC6, PC7, PC8, and PC9 described only a minor part (12.94%, 2.67%, 0.58%, 0.29%, 0.23%, 0.06%, 0.004%, and 0.002%, respectively) of total variation of the changes in RJQ-dependent variables. Their explanation is too low to take PC 2–9 into consideration in the subsequent analysis. Figure 3 shows that athletes treated with RJQ are grouped by the first two principal components into a separate cluster compared to PLA.

**FIGURE 4:**
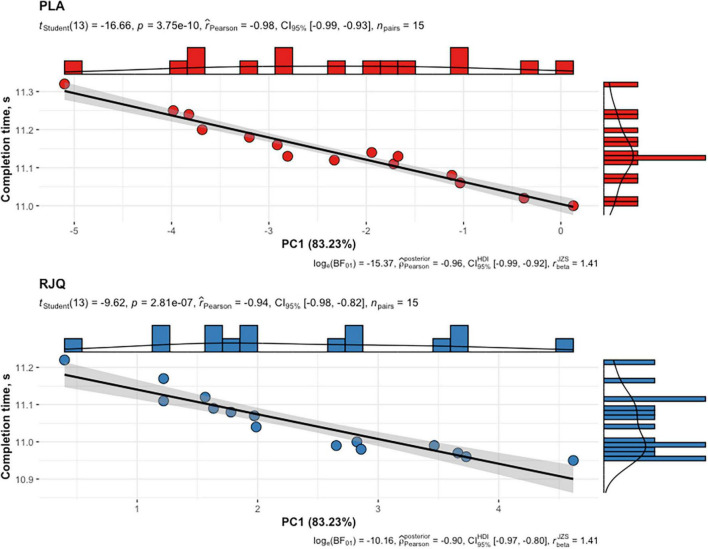
Scattergram of PC1 values and time taken by athletes to complete HIIE in both intervention groups. *n* = 15 per group. PC1, principal component 1; RJQ, royal jelly plus coenzyme Q10; PLA, placebo.

PC1 values in the RJQ group were more than those in the PLA group, which corresponded to a much lower increase in HR, SI, and blood lactate concentration in response to HIIE, as well as to the higher SDNN, RMSSD, pNN50, HF, LF, and VLF following exercise. It should be noted that there was a strong negative correlation between the time taken to complete HIIE and PC1 values in both intervention groups (Figure 4).

## Discussion

To our knowledge, this is one of the first randomized, placebo-controlled, double-blind trials to determine whether RJQ supplementation exerts beneficial effects on energetic metabolism and autonomic nervous system in athletes under HIIE conditions, and also on their exercise performance. The results of this study have shown that oral RJQ administration for 10 days was associated with a much lower increase in blood lactate concentration in response to the HIIE. The RJQ supplementation also induced a shift toward greater parasympathetic tone early at post-exercise, as indicated by lower HR and higher SDNN, RMSSD, and pNN50 compared to PLA. In addition, HRV frequency-domain parameters, such as HF power, LF power, and VLF power, in the RJQ group were higher than those in the PLA group, reflecting not only enhanced parasympathetic and sympathetic modulation but also increase in favor of baroreflex input and humoral influence on the heart. Moreover, the RJQ-induced changes in HRV and blood lactate concentration led to a shorter time taken by athletes to complete HIIE compared to PLA. Direction and strength of statistical relationship between the completion time and PC1 values demonstrate that the improvements in runners’ HIIE performance were due in significant part to RJQ-induced reduction of increment in blood lactate levels in response to exercise in combination with a more rapid shift in autonomic activity toward increased parasympathetic control early following exercise. Our prior work partially confirms these findings where 10-day RJQ ingestion increased HIIE performance in swimmers while reducing the increment in SI and blood lactate levels in response to exercise (33). And, unlike the present study, specific exercise protocol related to swimming was applied. Thereby, developing new pharmacological strategies that include RJQ supplementation could be potentially beneficial for improving HIIE performance in athletes and reducing the risk of adverse effects of increased intramuscular acidity and prolonged sympathetic dominance following intense exercise.

It is known that RJ and CoQ10 are able to promote ATP synthesis *via* glycolysis and oxidative phosphorylation (OXPHOS) (24, 25), respectively, suggesting that RJQ may play an important role in enhancing exercise performance and reductions in lactic acidosis. 10-hydroxy-2-decenoic acid (10H2DA), which is a unique fatty acid specifically found in RJ, has been reported to induce AMPK activation in skeletal muscle, leading to insulin-independent translocation of glucose transporter (GLUT) 4 to the plasma membrane and greater glucose uptake (21). In addition to 10H2DA, leucine and casein peptide found in RJ have been shown to also activate AMPK in skeletal muscles (23–25). Furthermore, AMPK, known to be activated not only by RJ but also by CoQ10, is responsible for the stimulation of glycolysis through phosphorylation of 6-phosphofructo-2-kinase that catalyzes the synthesis and degradation of fructose-2,6-bisphosphate, which in turn regulates the activity of 6-phosphofructo-1-kinase, thereby controlling the glycolysis rate (34–37). During very intense efforts lasting seconds, such as the 100-m sprint, most ATP is derived from the breakdown of phosphocreatine (PCr) and glycogen to lactate (16). OXPHOS is also activated but its contribution to ATP turnover during maximal exercise lasting about 12 s is low and may be highest after the duration of high-intensity interval exercise (repeated bouts of 100-m sprint with active recovery of 45 s between sets) extends beyond approximately 1 min (16, 38, 39). Despite the activation of the oxidative pathways in skeletal muscle during HIIE, the rates of ATP provision from the anaerobic sources (PCr and anaerobic glycolysis) are much more rapid than those from aerobic pathways, which resulted in the production of lactate and its subsequent accumulation in muscle and blood (16, 40). In this situation, CoQ10 contained in RJQ could stimulate mitochondrial OXPHOS through activation of complex I that regulates the rate in overall respiration (41). CoQ10, an essential cofactor in OXPHOS, is able to increase cellular ATP production, carrying the electrons from complexes I and II to complex III of the mitochondrial respiratory chain (23–25). On the other hand, CoQ10 might also indirectly increase the flux of glucose through pyruvate dehydrogenase (PDH) into the tricarboxylic acid cycle, in part *via* a decrease in NADH and possibly acetyl-CoA, which would keep PDH active during HIIE (42, 43). Therefore, glycolytic flux, activity of complex I and NAD^+^-dependent enzymes, and ultimately aerobic ATP production might be higher during exercise in athletes treated with RJQ compared to PLA. Perhaps, RJQ supplementation links the glycolytic pathway and OXPHOS in muscle cells, leading to greater ability to rapidly resynthesize PCr when the exercise intensity falls and athletes rest.

Interestingly, phosphorylation and activation of AMPK by 10H2DA are mediated *via* extracellular Ca^2+^/calmodulin-dependent protein kinase β (CaMKKβ) independently of changes in adenine nucleotides and liver kinase B1 pathway (21). Thus, 10H2DA-induced AMPK activation in response to a transient elevation of intracellular Ca^2+^ caused by muscle contractions appears likely to play an important role in regulating AMPK under exercise conditions. Despite this activation of AMPK in skeletal muscle, CaMKKβ-induced activation of AMPK is suggested to be a key pathway in neural tissue because expression of CaMKKβ is much higher in the brain than in other organs (44, 45). Novel biological contexts in which AMPK activation by calcium might be relevant are constantly being reported (46). One such notable context may be an activation of transient receptor potential ankyrin 1 (TRPA1) and vanilloid 1 (TRPV1), both of which are Ca^2+^-permeable non-selective cation channels (47, 48). Specific hydroxy fatty acids in RJ, such as 10H2DA, 10-hydroxydecanoic acid, have been shown to activate both TRPA1 and TRPV1, but TRPA1 more strongly than TRPV1 (20). Additionally, dicarboxylic acids showed equal efficacy for both channels, but they are present in only small amounts in RJ (20). The RJQ-induced activation of TRPA1 and TRPV1 appears to have a role in the regulation of both sympathetic and parasympathetic branches of the autonomic nervous system, likely in a biphasic manner characterized by early sympathetic activation, when exercise begins, followed by a shift toward greater vagal dominance as rapidly as needed, when transitioning from exercise to rest occurs or the exercise intensity falls to low levels. Activation of TRPA1 in muscle afferent nerves has been shown to amplify sympathetic responsiveness *via* the metabolic component of the exercise pressor reflex when blood supply to the muscles is insufficient, which occurs during HIIE (49). In contrast, blocking TRPA1 has been reported to attenuate sympathetic nerve activity response evoked by muscle contraction and not to affect sympathetic modulation during passive tendon stretch (49). Similarly, TRPV1 channel is involved in the skeletal muscle exercise pressor reflex (50). Smith et al. found that TRPV1 blockade alleviates the increase in heart rate and blood pressure during muscle contraction (51). However because we did not measure HRV parameters during transitions from rest to exercise in the present study, we cannot confirm the presence of enhanced sympathetic drive during exercise in the RJQ group compared to the PLA group. Instead, we observed opposite effects of RJQ supplementation on HRV during the transition from HIIE to recovery and concluded that the parasympathetic dominance (i.e., increased SDNN, RMSSD, pNN50, and HF power) may be in part due to RJQ-induced reductions in oxidative stress caused by exercise (26). Our previous study has suggested that improved high-intensity interval exercise performance in swimmers is partially associated with the RJQ-induced reducing in lipid peroxidation and muscle damage during exercise (52). It should be noted that exposure to exogenous activators of TRPA1 and TRPV1 has been shown to cause a slight increase in parasympathetic influence on heart rate in healthy subjects at rest (53). Importantly, TRPV1 activation improves baroreflex sensitivity and energy metabolism (50, 54). Since LF power and VLF power reflect baroreflex function and energy metabolic regulation, respectively (53, 55), the possible activation of TRPV1 by RJQ may be an explanation for significantly higher values of these HRV frequency-domain parameters in athletes treated with RJQ compared to PLA under exercise conditions. Besides, Luo et al. revealed that chronic TRPV1 activation induces expression of the transcriptional cofactor peroxisome proliferator-activated receptor-γ coactivator-1α (PGC-1α) and enhances mitochondrial function in a Ca^2+^-dependent manner in the skeletal muscle, leading to greater exercise endurance (56). Increased HIIE performance in participants who received RJQ compared to PLA for 10 days is likely to be TRPA1/TRPV1-mediated Ca^2+^-signaling mechanisms that involve CaMKKβ and AMPK. Of note, RJQ-induced AMPK activation may be functionally more important for the post-exercise changes in muscle metabolism and insulin sensitivity, and for mediating some of the key adaptive responses to exercise in skeletal muscle, such as mitochondrial biogenesis, enhanced antioxidant protection, and increased GLUT4 expression. AMPK increases mitochondrial biogenesis in skeletal muscle *via* activation of PGC-1α that enhances mitochondrial tissue content and subsequently the capacity of the cell to respond to future energetic challenges (46, 57). Therefore, the long-term changes induced by RJQ supplementation may be associated with improved skeletal muscle fiber transformation from glycolytic type II fibers to more oxidative type I fibers and, consequently, increased respiratory function. It is known that type I fibers have more mitochondrial mass and produce more ATP from lipid oxidation but less from glycolysis than type II fibers, thus providing stable energy state for a longer time with less lactate accumulation (56, 58–60). A recent study demonstrated that RJ treatment with endurance training induced mitochondrial adaptation through activation of AMPK in mice soleus muscle (22), which predominantly consists of type I and type IIA muscle fibers (61–63). These findings support the hypothesis that oral ingestion of RJQ for 10 days in conjunction with regular exercise might yield synergies toward greater HIIE performance through multiple complementary pathways, as stated above.

This study has some limitations that should be noted. First, the CoQ10 levels in blood plasma and skeletal muscle were not quantified. The second limitation derives from the origin of the sample. The fact that all athletes were associated with sprinting and recruited from a single sports center prompts us to be cautious in generalizing the results to athletes who perform HIIE and participate in other sports. Additional context that may be considered as important for the RJQ supplementation is its dose and timing, which were pre-established, and the dose-response effect could not be analyzed in the present study. It may be useful to consider higher dosages and longer interventions to determine their potential benefit. Nevertheless, our study had important strengths because there are no studies, excluding our findings, which have analyzed RJQ as a nutritional supplement in athletes. Further studies with athletes who perform various types of muscular exercise are now needed to determine whether RJQ supplementation may be effective not just in increasing runners’ HIIE performance but also in other sport-specific performances.

## Conclusion

The study reveals that RJQ supplementation significantly reduced the exercise-induced increment in blood lactate concentration in combination with a more rapid shift in autonomic balance toward increased parasympathetic control early following exercise compared to PLA. We also found a strong negative correlation between the time taken to complete HIIE and PC1 values, which described more than 83% of the total variance in HR, SDNN, RMSSD, pNN50, HF, LF, VLF, SI, and blood lactate. An important point to note here is that PC1 values in the RJQ group were more than those in the PLA group, which corresponded to a much lower increase in HR, SI, and blood lactate concentration in response to HIIE, as well as to higher SDNN, RMSSD, pNN50, HF, LF, and VLF following exercise. Thus, we demonstrated that RJQ-induced changes in cardiac autonomic regulation and blood lactate levels in runners under HIIE conditions enhance their exercise performance.

## Data Availability Statement

The raw data supporting the conclusions of this article will be made available by the authors, without undue reservation.

## Ethics Statement

The studies involving human participants were reviewed and approved by the Bioethics Committee of Lobachevsky University (approval number: 43). The participants provided their written informed consent to participate in this study.

## Author Contributions

AO: conceptualization and visualization. AO and AD: formal analysis, investigation, resources, and data curation. AO and AP: writing-original draft preparation. AO, AD, and AP: writing-review and editing. All authors have read and agreed to the published version of the manuscript.

## Conflict of Interest

The authors declare that the research was conducted in the absence of any commercial or financial relationships that could be construed as a potential conflict of interest.

## Publisher’s Note

All claims expressed in this article are solely those of the authors and do not necessarily represent those of their affiliated organizations, or those of the publisher, the editors and the reviewers. Any product that may be evaluated in this article, or claim that may be made by its manufacturer, is not guaranteed or endorsed by the publisher.
